# Membrane-bound O-acyltransferase 7 (MBOAT7)-driven phosphatidylinositol remodeling in advanced liver disease

**DOI:** 10.1016/j.jlr.2022.100234

**Published:** 2022-05-27

**Authors:** Venkateshwari Varadharajan, William J. Massey, J. Mark Brown

**Affiliations:** 1 Department of Cardiovascular and Metabolic Sciences, Lerner Research Institute Cleveland Clinic, Cleveland, OH, USA; 2 Center for Microbiome and Human Health, Lerner Research Institute, Cleveland Clinic, Cleveland, OH, USA

**Keywords:** phospholipid, fibrosis, fatty liver, diabetes, obesity, nonalcoholic fatty liver disease, alcohol-associated liver disease, AA, arachidonic acid, ALD, alcohol-associated liver disease, ASO, antisense oligonucleotide, COVID-19, coronavirus disease 2019, CDP-DAG, CDP-diacylglycerol, CDS2, CDP-diacylglycerol synthase 2, DGLA, di-homo-γ-linolenic acid, FATP1, fatty acid transport protein 1, GPR55, G protein-coupled receptor 55, GWAS, genome-wide association study, HBV, hepatitis B virus, HCC, hepatocellular carcinoma, HCV, hepatitis C virus, LPI, lysophosphatidylinositol, *MBOAT7*, membrane-bound O-acyltransferase 7, *Mboat7*^HKO^, *Mboat7* hepatocyte-specific knockout mice, MPO, morpholino oligonucleotide, NAFLD, nonalcoholic fatty liver disease, NASH, nonalcoholic steatohepatitis, PA, phosphatidic acid, PC, phosphatidylcholine, PE, phosphatidylethanolamine, PI, phosphatidylinositol, PIP, phosphatidylinositol phosphate, PLA_2_, phospholipase A2, *Scap*, SREBP cleavage-activating protein

## Abstract

Advanced liver diseases account for approximately 2 million deaths annually worldwide. Roughly, half of liver disease-associated deaths arise from complications of cirrhosis and the other half driven by viral hepatitis and hepatocellular carcinoma. Unfortunately, the development of therapeutic strategies to treat subjects with advanced liver disease has been hampered by a lack of mechanistic understanding of liver disease progression and a lack of human-relevant animal models. An important advance has been made within the past several years, as several genome-wide association studies have discovered that an SNP near the gene encoding membrane-bound *O*-acyltransferase 7 (*MBOAT7*) is associated with severe liver diseases. This common *MBOAT7* variant (rs641738, C>T), which reduces MBOAT7 expression, confers increased susceptibility to nonalcoholic fatty liver disease, alcohol-associated liver disease, and liver fibrosis in patients chronically infected with viral hepatitis. Recent studies in mice also show that *Mboat7* loss of function can promote hepatic steatosis, inflammation, and fibrosis, causally linking this phosphatidylinositol remodeling enzyme to liver health in both rodents and humans. Herein, we review recent insights into the mechanisms by which MBOAT7-driven phosphatidylinositol remodeling influences liver disease progression and discuss how rapid progress in this area could inform drug discovery moving forward.

The liver plays an essential role in human health, serving as the central organizing center for the metabolism of the diet that we eat and the xenobiotics to which we are exposed. Given its central role in metabolism and drug detoxification, liver failure is not compatible with life. Advanced liver diseases such as cirrhosis or hepatocellular carcinoma (HCC) can be driven by a variety of initiating factors, including infectious agents (hepatitis A, B, and C), excessive exposure to certain drugs or toxins (i.e., dioxins, acetaminophen, aflatoxins, etc), inborn errors of metabolism (hemochromatosis, Wilson’s disease, alpha-1 antitrypsin deficiency, etc), autoimmune conditions (autoimmune hepatitis, primary sclerosing cholangitis, primary biliary cholangitis, etc), heavy alcohol use (alcohol-associated liver disease [ALD]), or obesity and diabetes-related factors in those who do not drink alcohol (nonalcoholic fatty liver disease [NAFLD]). Although we have identified the diverse paths leading to end-stage liver disease, we still have an immature understanding of the molecular mechanisms driving etiology-specific disease progression. It is clear that interactions between genetic determinants and environmental factors combine to facilitate liver disease progression, and by understanding these interactions, there is hope that new therapeutic strategies can be realized. Currently, the only treatment option for those suffering from end-stage liver disease is liver transplantation. However, the number of donor livers needed far surpasses the supply, which unfortunately means that being diagnosed with advanced liver disease is nearly synonymous with receiving a death sentence. Therefore, further understanding of the molecular mechanisms underlying the progression of liver disease from simple steatosis to more advanced inflammatory and fibrotic disease could have a broad impact in all forms of liver disease.

Recently, a new lipid metabolic pathway has emerged as a key driver of liver disease progression across viral and nonviral etiologies. Since late 2015, a growing number of genetic association studies have identified a loss-of-function variant (rs641738, C>T) near the gene encoding membrane-bound *O*-acyltransferase 7 (*MBOAT7*). This gene encodes a lysophospholipid acyltransferase enzyme (lysophosphatidylinositol [LPI] acyltransferase 1), which plays a very unique role in selectively diversifying the PUFA composition of phosphatidylinositols (PIs) at the nucleophilic substitution 2 (*sn*-2) position. In this review, we discuss the growing number of human genetic studies linking *MBOAT7* to liver disease and other neurological disorders (Table 1), the biochemical and physiologic role of MBOAT7-driven PI remodeling, and our current understanding of the molecular mechanisms by which *MBOAT7* loss-of-function predisposes to liver injury.

**Table 1:** Human studies linking MBOAT7 function to diverse human disease

Disease etiology	Study information	Major findings	References
ALD	GWAS for alcohol-related cirrhosis in individuals of European descent (712 cases and 1,426 controls) with subsequent validation in two independent European cohorts (1,148 cases and 922 controls)	rs641738 variant near *MBOAT7* is associated with alcohol-related cirrhosis	Buch *et al.* (1)
ALD	Genetic association study for HCC (765 Italian liver disease patients)	rs641738 variant is not significantly associated with alcohol-related HCC	Stickel *et al.* (3)
ALD	Large case-control multicenter study for SNP in association with ALD (507 ALD patients and 645 ethnically matched healthy controls—Han Chinese population)	rs641738 variant is not associated with indices of liver damage in alcohol users	Zhang *et al.* (2)
ALD	Effect of *MBOAT7* rs641738 variant on AH and severity (211 patients with AH and 176 heavy drinking controls)	rs641738 variant did not show significant relationships with AH	Beaudoin *et al.* (4)
NAFLD	Population based (first stage); cases only (second stage) associated with steatosis, NASH, fibrosis stage (3,854 participants from the Dallas Heart Study [first stage]; 1,149 cases from LBC [second stage] from European descent)	rs641738 variant was associated with an increased hepatic fat content, more severe liver damage, and increased risk of hepatic fibrosis	Mancina *et al.* (8)
NAFLD	Genetic variation in *MBOAT7* associated with steatosis, NASH, fibrosis stage (125 cases)	rs641738 variant associates with histologic liver damage, particularly significant fibrosis	Luukkonen *et al.* (9)
NAFLD	Hospital-based prospective cohort investigating relationship between MBOAT7 and outcomes of bariatric surgery (84 obese individuals)	*MBOAT7* might regulate not only hepatic fat accumulation but also the whole-body adiposity	Krawczyk *et al.* (10)
NAFLD	Investigating rs641738 near *MBOAT7* modulate both steatosis and fibrosis in NAFLD patients (multicenter biopsy-based study—515 patients with NAFLD)	rs641738 variant was linked with increased hepatic fat content, severe liver disease, and increased risk of fibrosis	Krawczyk *et al.* (11)
NAFLD	*MBOAT7* variant in association with NAFLD and liver injury	rs641738 variant was more frequently associated with severe hepatic steatosis and to a lesser extent with NAFLD risk and liver injury modulation	Krawczyk *et al.* (12)
NAFLD/HCC	rs626283 polymorphism in the *MBOAT7* associated with increased risk of MAFLD-HCC and alcohol-related or HCV-related HCC (765 noncirrhotic MAFLD cases [HCC, *n* = 132]); 1,121 noncirrhotic patients affected by ALD or HCV (HCC, *n* = 25)	MBOAT7 loss of function is independently associated with HCC risk	Donati *et al.* (13)
NAFLD	A Mendelian randomization approach to examine whether hepatic fat causally determines liver damage and metabolic comorbidities (liver biopsy cohort involving 1,515 individuals, the Swedish Obese Subjects Study involving 3,329 subjects, and the Dallas Heart Study with 4,570 participants)	rs641738 variant exerted significant effects on hepatic fat, liver damage, and metabolic traits	Dongiovanni *et al.* (14)
NAFLD	GWAS for NAFLD; resequencing strategy by next-generation sequencing in a cohort of 218 NAFLD subjects and 227 controls	rs641738 variant is associated with NAFLD and possibly influencing its severity	Di Costanzo *et al.* (15)
NAFLD	To explore the effect derived from silybin-phospholipid complex, oral administration in NAFLD patients carrying *MBOAT7*-rs641738 variant (92 biopsy-proven NAFLD patients were grouped in 30 NAFLD wild-type controls, 30 wild-type-treated patients, and 32 mutated treated ones)	The assessed mutations are independently associated with no response to a silybin-based therapeutic regimen and could be considered as useful predictive markers	Dallio *et al.* (16)
ALD/NAFLD	Polygenic risk score on genetic variants in *MBOAT7* associated with severe liver disease (266,687 individuals in the UK Biobank)	rs641738 variant was causally related to liver injury and strongly associated with severe liver disease	De Vincentis *et al.* (17)
NAFLD/HCC	Learning NAFLD cohort, *n* = 2,566; 226 with HCC; and a replication cohort of 427 German patients with NAFLD and the general population (UK Biobank [UKBB] cohort, *n* = 364,048; 202 with HCC)	rs641738 variant is associated with hepatic fat, and hepatic fat is causally related to HCC	Bianco *et al.* (18)
Pediatric NAFLD	Longitudinal follow up in 467 Caucasian children aged 6–9 years old	rs641738 T allele had higher plasma ALT levels	Viitasalo *et al.* (19)
Pediatric NAFLD	rs641738 variant near *MBOAT7* associated with NAFLD (1,002 Italian obese children and adolescents)	The rs641738 variant in obese children showed elevated serum level of ALT. This is the first pediatric association of the *MBOAT7* polymorphism with indirect markers of liver fibrosis	Di Sessa *et al.* (22)
Pediatric NAFLD	Multiethnic cohort of obese children and adolescents, we genotyped the rs626283 polymorphism in the *MBOAT7* gene	The rs626283 variant is associated with NAFLD and altered glucose metabolism	Umano *et al.* (23)
NAFLD	Case-control hospital-based cohort study on NAFLD (634 individuals; 372 patients with NAFLD diagnosed by liver biopsy and 262 control subjects)	The rs641738 variant is not associated with NAFLD or the histological disease severity	Sookoian *et al.* (24)
NAFLD	Case-control hospital-based cohort to study the *MBOAT7* association with NAFLD (416 cases and 109 controls)	No association between the rs641738 variant and any of the histological severity markers of NAFLD	Koo et at. (25)
NAFLD	To study the effect of *MBOAT7* rs626283 variant on renal function and NAFLD (prospective Asian cohort from NAFLD registry)	Rs641738 variant is associated with CKD mediated by increased systemic inflammation	Koo *et al.* (26)
NAFLD	453 patients with biopsy-proven NAFLD with sufficient clinical data for calculating scores (*n* = 302; discovery cohort; *n* = 151; validation cohort)	rs641738 variant is not associated with NAFLD or NASH phenotypes	Koo *et al.* (27)
NAFLD/cirrhosis	Study of an Eastern European population that assessed the impact of *MBOAT7* rs641738 on developing liver injury (1,012 individuals)	rs641738 variant was not linked to hepatic fibrosis, alcohol, or hepatitis C virus-induced liver cirrhosis in an Eastern European population	Basyte-Bacevice *et al.* (28)
NAFLD	958 middle-aged Finns, 249 with NAFLD, were followed for 21 years	rs641738 variant not associated with overall mortality associated with the metabolic syndrome	Karajamki *et al.* (29)
NAFLD	294 patients (63% women) with a mean age of 53 (±17) years and 31% Hispanic ethnicity with genotyping	rs641738 variant was not associated with advanced fibrosis	Ajmera *et al.* (30)
NAFLD	GWAS involved 1,483 biopsied NAFLD cases and 17,781 controls	rs641738 variant was not associated with NAFLD	Anstee *et al.* (31)
Pediatric NAFLD	Genetic variant, demographic, and biochemical data analysis on the effects on NAFLD (126 enrolled subjects, 84 in the case group and 42 in the control group)	rs641738 variant is not a risk factor for NAFLD in obese US children of Hispanic ethnicity	Mansoor *et al.* (32)
Pediatric NAFLD	Association of *MBOAT7* rs641738 variant and NAFLD in the pediatric population (1,760 overweight or obese children)	No significant contribution of the rs641738 variant to the risk of NAFLD/NASH in a large hospital-based cohort of Italian overweight/obese children	Zusi *et al.* (33)
Pediatric NAFLD	rs641738 genotyping in 232 children with obesity and NAFLD	No significant contribution of the rs641738 variant to the risk of NAFLD	Di Costanzo *et al.* (34)
Pediatric NAFLD	831 obese children aged 7–15 were genotyped for the rs641738 variant	rs641738 variant was not associated with hepatic steatosis or CK-18 fragment in obese Taiwanese children	Lin *et al.* (35)
NAFLD	Meta-analysis of *MBOAT7* associated with steatosis, MAFLD severity, fibrosis stage, and HCC (42 studies, including 1,047,265 participants [including 4,174 children], out of which 7,692 had liver biopsy and 45,419 had minor T allele of rs641738 C>T)	rs641738 variant was associated with elevated hepatic steatosis and is linked to more severe fatty liver disease	Teo *et al.* (36)
HCV	Study associated with severe hepatic inflammation and increased risk of fibrosis (2,051 Caucasian consecutive subjects, including 1,706 with CHC, 931 in the discovery cohort, and 775 in the validation cohort; 270 healthy controls and 75 with HCV-related HCC)	rs641738 variant is a novel risk variant for liver inflammation in hepatitis C and thereby for liver fibrosis	Thabet *et al.* (44)
HBV	Functional analysis associated with hepatic inflammation and fibrosis in chronic hepatitis B (1,101 HBV cases)	rs641738 variant associated with hepatic inflammation and fibrosis in patients with HBV	Thabet *et al.* (45)
HBV/HCV	Association between the *MBOAT7* rs641738 polymorphism and disease progression of HCV and HBV infection (971 consecutive Moroccan subjects [288 with CHC, 98 formerly HCV-infected patients, 268 with CHB, 126 HBV, and 191 healthy controls])	rs641738 variant is not associated with progression of liver disease in chronic HBV or HCV	Ezzikouri *et al.* (46)
HBV/HCV/HCC	rs641738 was genotyped in 105 healthy controls and 530 patients with HCC (270 with HBV, 131 with HCV) and 129 with no virus detected matched for age and gender	rs641738 variant was not associated with HCV- or HBV-associated HCC	Raksayot *et al.* (47)
HCV/HCC	About 56 patients with HCV-associated cirrhosis who underwent antiviral therapy	ra641738 variant was not associated with HCV associate with response to therapy	Dunn *et al.* (48)
HCV/HCC	A total of 171 patients who received direct acting antiviral therapy	rs641738 variant was not associated with response to therapy	Kang *et al.* (49)
HBV/HCC	Case-control study for *MBOAT7* rs641738 in the risk of HCC and persistent HBV infection (779 HCC cases and 1,412 cancer-free controls)	rs641738 variant is not associated with the risk of HCC or persistent HBV infection	Wang *et al.* (50)
PSC	Case study of two *MBOAT7* variant alleles (TT and CT) on PSC patients (262 PSC cases from Freund 2020 study and 252 patients with PSC from Rahal 2020 study)	Liver transplant-free survival was significantly prolonged in carriers of two rs641738 variant allele	Freund *et al.* (51), Rahal *et al.* (52)
Hemochromatosis	rs641738 was genotyped in 1,319 C282Y homozygotes, from six European countries, of whom 171 (13.0%) had cirrhosis	rs641738 variant was not associated with hemochromatosis outcome	Buch *et al.* (53)
CVD	GWAS of 141 lipid species (*n* = 2,181 individuals), phenome-wide scans with 25 CVD-related phenotypes (*n* = 511,700 individuals)	*MBOAT7* rs8736 variant not associated with CVD mortality, and TT carriers showed significantly reduced levels of PI (18:0;0–20:4;0)	Tabassum *et al.* (54)
CVD	Meta-analysis of 48 GWAS studies for CAD (60,801 CAD cases; 123,504 controls)	rs641738 variant had neutral effects in coronary artery disease	Simons *et al.* (55), Brouwers *et al.* (56)
Gastric cancer	A fine-mapping association study in 1,926 gastric cancer patients and 2,012 controls of European descent	Downregulation of MBOAT7 expression is associated with gastric cancer risk	Heinrichs *et al.* (57)
Lung cancer	Study of MBOAT7 function non-small-cell lung cancer cell lines	*MBOAT7* is necessary for proliferation and in vivo tumor formation in mice	Saliakoura *et al.* (58)
Kidney cancer	Study of MBOAT7 function in clear cell renal carcinoma (ccRCC) cell lines	*MBOAT7* is overexpressed in ccRCC, and *MBOAT7* knockout prevents in vivo tumor formation	Neumann *et al.* (59)
Intellectual disability	Sequencing on individuals with intellectual disability and other neurological conditions	Inactivating variants in *MBOAT7* lead to intellectual disability accompanied by epilepsy and autistic features in patients	Johansen *et al.* (60), Jacher *et al.* (61), Khan *et al.* (62), Heidari *et al.* (63), Yalnizoglu *et al.* (64)
COVID-19	Prospectively analyzed a cohort of 44 patients with COVID-19	rs641738 genotype: only *n* = 8 were wild-type CC, and the remaining *n* = 36 were carrying the variant allele (19 heterozygous and 17 homozygous carriers)	Machill *et al.* (65)

AH, alcoholic hepatitis; ALT, alanine aminotransferase; CHC, combined hepatocellular cholangiocarcinoma; CKD, chronic kidney disease; CVD, cardiovascular disease; MAFLD, metabolic-associated fatty liver disease; PSC, primary sclerosis cholangitis.

## Genome-wide association studies identify *MBOAT7* as a risk locus for ALD

**Fig. 1:**
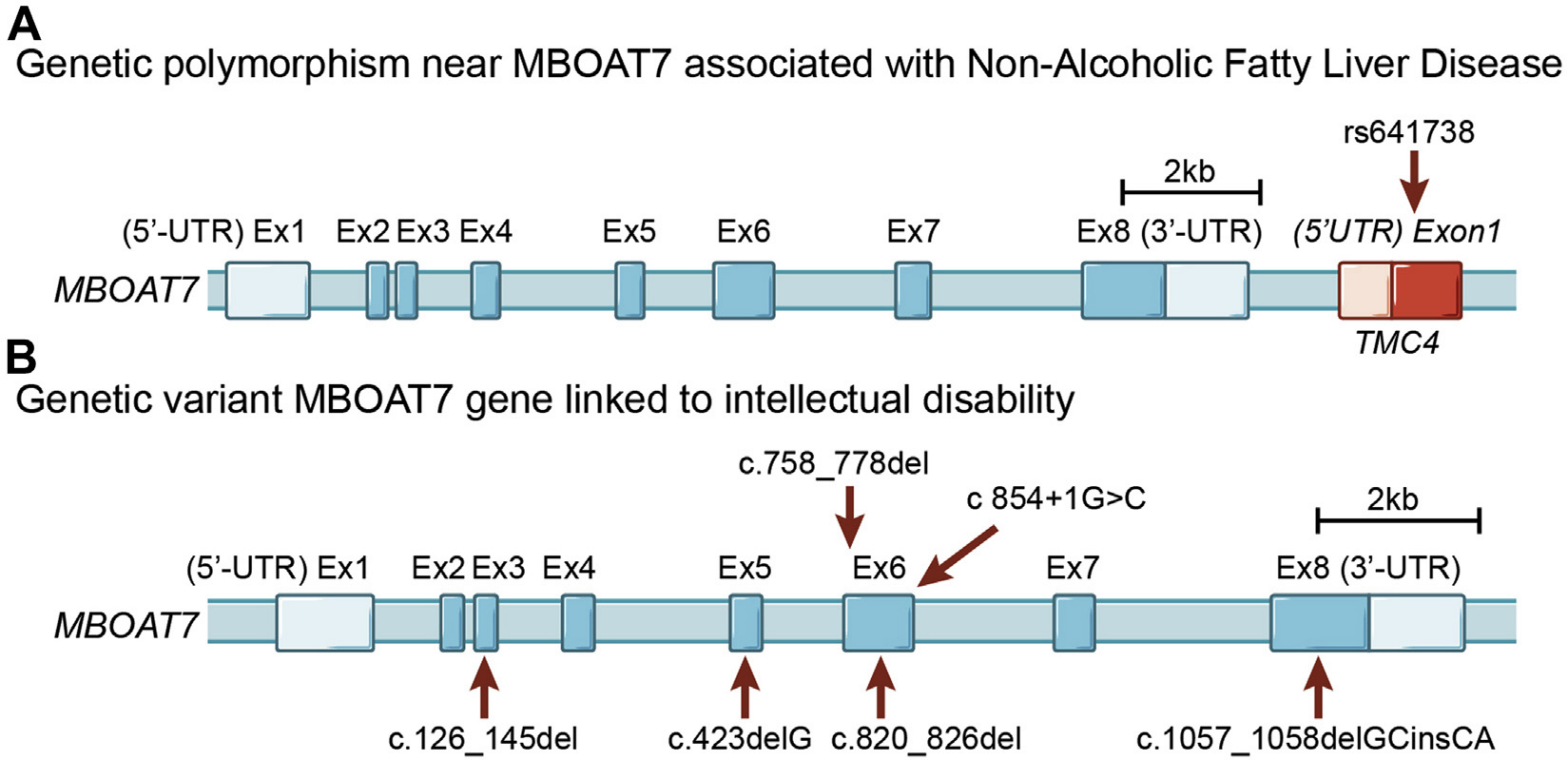
Genetic variation in *MBOAT7* is associated with diverse liver diseases and neurodevelopmental disorders. A: Genetic structure of the *MBOAT7* locus including the first exon of the neighboring gene transmembrane channel 4 (*TMC4*). The common rs641738 SNP (C>T) associated with diverse liver diseases including non-NAFLD is in close proximity to the 3′-untranslated region (3′-UTR) of *MBOAT7* but actually resides in exon 1 of the *TMC4* gene. B: Several functionally null mutations in MBOAT7 have been associated with severe intellectual disability, epilepsy, and autistic features (Online Mendelian Inheritance in Man: 606048). Variants are indicated by red arrows.

The first study to associate *MBOAT7* variants with advanced liver disease was published in late 2015 by Buch *et al.* (1). Excessive alcohol intake is the leading cause of cirrhosis in developed countries, so this group initially performed a genome-wide association study (GWAS) in a learning cohort of 712 cases of alcohol-associated cirrhosis and 1,426 controls of European descent and made the initial observation that rs641738 (C>T) variant was associated with increased risk of alcohol-related cirrhosis. This finding was confirmed in an independent validation cohort of 1,148 cases and 922 controls consistently linking the *MBOAT7* variant with alcoholic cirrhosis (*P* = 1.03 × 10^−9^) (1). Buch *et al.* also performed *cis* expression quantitative trait locus analysis and showed that the rs641738 variant was associated with reduced gene expression of *MBOAT7*, but not the neighboring gene *TMC4*, an observation that has been confirmed by many other studies to date (8, 24, 36, 37, 39). This initial *cis* expression quantitative trait locus was an extremely important observation given that the rs641738 locus is actually positioned in exon 1 of the *TMC4* beyond the 3′ untranslated region of *MBOAT7* (Fig. 1) and provided early evidence that the causal gene in the locus is *MBOAT7* and not *TMC4*. This seminal study by Buch *et al.* set the stage for other validation studies in ALD as well as other liver disease etiologies. It is important to note that an independent multicenter study of 507 ALD patients and 645 healthy controls in a Han Chinese population by Zhang *et al.* (2) did not find a statistically significant association between the rs641738 variant and indices of liver damage. Furthermore, unlike the association between rs641738 and alcohol-related cirrhosis (1), studies examining alcohol-related development of HCC have likewise not found a significant association (3). Also, the rs641738 variant is not significantly associated with the risk or severity of a condition known as alcoholic hepatitis, which is an acutely severe life-threatening condition traditionally seen in heavy binge drinkers (4). Collectively, the largest studies examining associations between the rs641738 variant and alcohol-associated liver injury support a strong association in alcohol-related cirrhosis in people of European descent (1), but additional large-scale studies are needed to see how generalizable this is across diverse populations.

## Genetic variation near *MBOAT7* confers increased susceptibility to the entire spectrum of NAFLD

Although the association between *MBOAT7* and ALD seems to be mostly confined to alcohol-related cirrhosis, there are now numerous large-scale studies linking the rs641738 locus to the entire spectrum of NAFLD, including simple steatosis, nonalcoholic steatohepatitis (NASH), cirrhosis, and HCC (5, 6, 7, 8, 9, 10, 11, 12, 13, 14, 15, 16, 17, 18, 19, 20, 21, 22, 23, 24, 25, 26, 27, 28, 29, 30, 31, 32, 33, 34, 35, 36, 37, 38, 39, 40, 41, 42, 43, 44, 45, 46, 47, 48, 49, 50, 51, 52, 53, 54, 55, 56, 57, 58, 59, 60, 61, 62, 63, 64, 65). NAFLD is rapidly becoming the leading cause of end-stage liver disease, closely paralleling the worldwide rise in obesity and type 2 diabetes mellitus (5, 6, 7, 8). Therefore, drug discovery in the NAFLD space has rapidly expanded over the past decade. Several genes encoding lipid metabolic regulators have been identified as NAFLD risk loci; the most reproducible being patatin-like phospholipase domain containing 3 (*PNPLA3*) and transmembrane 6 superfamily member 2 (*TM6SF2*) (5, 6, 7, 8). A landmark study in 2016 by Mancina *et al.* (8) identified the rs641738 variant as a susceptibility locus for NALFD in individuals of European descent. This study genotyped the rs641738 locus in 3,854 subjects in the multiethnic Dallas Heart Study as well as another validation cohort of 1,149 European subjects from the liver biopsy cross-sectional cohort. Importantly, these subjects had hepatic steatosis and other disease indices measured by either proton magnetic resonance spectroscopy or liver biopsy. In this key study, the rs641738 minor T allele was associated with increased hepatic steatosis and more severe liver damage including fibrosis (8). This study also confirmed lower MBOAT7 protein abundance by Western blotting and lower levels of the MBOAT7 enzymatic product (38:4 PI) in subjects with the minor allele (8). Since this original study by Mancina *et al.*, a large number of independent studies have confirmed and extended their original observation linking the rs641738 locus with NAFLD progression to fibrosis and HCC. Luukkonen *et al.* (9) examined liver biopsies from 125 insulin-resistant subjects and found that the rs641738 variant associates with histologically proven fibrosis and show that minor T-allele carriers have significantly less 38:4 PI in their liver. Krawczyk *et al.* (10) prospectively monitored 84 obese individuals before and after bariatric surgery and found that the rs641738 variant was associated with increased triglyceride, cholesterol, low-density lipoprotein cholesterol, and glucose levels but was not an independent predictor of NAFLD improvement postbariatric surgery (10). Another study by the same group published in the *Journal of Lipid Research* studied 515 subjects with NAFLD, with 320 having biopsy-proven disease (11). This study found that the rs641738 T variant was solely associated with fibrosis but not steatosis grade in NAFLD patients (11). Krawczyk *et al.* (12) also analyzed 63 biopsy-proven NAFLD subjects in the German NAFLD Clinical Study Group program and found that the rs641738 variant was more frequent in NAFLD subjects with severe hepatic steatosis and may facilitate liver injury in obese patients without diabetes. Aligned with the concept that MBOAT7 loss of function may facilitate the progression of NAFLD, Donati *et al.* (13) found that the rs641738 T allele is associated with increased HCC in noncirrhotic NAFLD patients. Dongiovanni *et al.* (14) performed a Mendelian randomization study using a polygenic risk score including *MBOAT7* and found that hepatic steatosis plays a causal role in the development of chronic liver disease and that the rs641738 is associated with steatosis. In an independent study, Di Costanzo *et al.* (15) found that the rs641738 T allele is associated with NAFLD severity. Dallio *et al.* (16) recently studied the impact of the rs641738 variant on the efficacy of a potentially therapeutic silybin-phospholipid complex in NAFLD patients and found some preliminary association with response to therapy in this randomized controlled trial. Furthermore, in several polygenic risk score prediction studies, the rs641738 can improve prediction in NAFLD outcomes (14, 15, 17, 18).

Although NAFLD is a chronic and progressive disease where end-stage manifestations such as cirrhosis and HCC are usually present in adults, there has been a striking increase in the prevalence of NAFLD in young children and adolescents (19, 20). Evidence is just now emerging that MBOAT7 loss of function may also be linked to pediatric NAFLD progression. Viitasalo *et al.* (21) were the first to show that children with the rs641738 T allele had higher plasma alanine aminotransferase levels, a marker of hepatocyte death. This finding was corroborated by Di Sessa *et al.* (22) showing that the carriers of the rs641738 T allele had higher plasma alanine aminotransferase levels and also increased fibrosis assessed by the pediatric NAFLD fibrosis index score. It is also interesting to note that Umano *et al.* (23) identified the rs626283 polymorphism in the *MBOAT7* gene, which is also associated with insulin resistance and fatty liver in obese children and adolescents. It is important to note that the rs626283 SNP is in strong linkage disequilibrium with rs641738 across numerous studies (23). Although the majority of large studies find a clear association between MBOAT7 loss of function and NAFLD progression, it is important to point out that not all studies have found statistically significant associations (24, 25, 26, 27, 28, 29, 30, 31, 32, 33, 34, 35). Although consistent trends in association exist, several independent studies looking at NAFLD-related indices in adults (24, 25, 26, 27, 28, 29, 30, 31) and children (32, 33, 34, 35) have failed to conclude that the rs641738 SNP is associated with NAFLD progression. However, it is important to consider that a recent meta-analysis of all published studies including over 1 million subjects confirmed that the rs641738 variant is a risk factor for the presence and severity of NAFLD in individuals of European descent (36). These recent genetic studies support the notion that therapeutic strategies impacting MBOAT7 could be potentially useful in treating NAFLD, a concept that is also supported by recent animal studies in *Mboat7*-deficient mice described in detail below (37, 38, 39, 40, 41, 42).

## Genetic variation near *MBOAT7* confers increased susceptibility to viral hepatitis-induced fibrosis and liver cancer

Although the prevalence of both ALD and NAFLD is rapidly rising in developed countries, worldwide end-stage liver diseases such as cirrhosis and HCC are more commonly initiated by a viral infection, especially by chronic hepatitis B virus (HBV) and hepatitis C virus (HCV) (42, 43). Thabet *et al.* (44) first described the genetic associations between *MBOAT7* and both HBV and HCV. First, this group studied a cohort of 2,051 HCV patients and found that the rs641738 T allele was associated with severe hepatic inflammation, increased risk of developing fibrosis, and increased rate of fibrosis progression. This study also made important observations showing that the rs641738 allele is linked to reduced *MBOAT7* messenger RNA and protein in the blood and liver, and circulating markers of oxidative stress, macrophage activation, and increased inflammation (44). This same group also studied a cohort of 1,101 patients with chronic HBV infection and found that although the well-known SNPs in *PNPLA3* and *TM6SF2* were not associated, the rs641738 variant near *MBOAT7* was significantly associated with greater hepatic inflammation (45). Despite these original reports, several independent studies with smaller sample sizes have failed to find a statistically significant association between the rs641738 locus and HBV- or HCV-induced liver disease progression (46, 47, 48, 49, 50).

## Other links between MBOAT7 function and human disease

Although the most striking association to date with the rs641738 locus track with NAFLD progression, there have been several other recent studies providing important new clues into potential links between MBOAT7 function and diseases of the liver and in extrahepatic organs. Another rare chronic liver disease associated with narrowing of the bile ducts called primary sclerosis cholangitis can often result in liver cirrhosis and ultimately to liver failure. Contrary to its pathological association in the aforementioned liver diseases, a recent study by Freund *et al.* (51) found that the rs641738 T allele is surprisingly associated with prolonged transplant-free survival in primary sclerosis cholangitis patients. This study also found that *MBOAT7* is highly expressed in biliary epithelial cells as well as hepatic stellate cells that are critical for profibrotic collagen deposition (51, 52). Furthermore, cirrhosis in subjects with hereditary hemochromatosis does not appear to be genetically linked to *MBOAT7* (53). Fatty liver disease is often associated with hyperlipidemia because of the overproduction of hepatic very low-density lipoproteins. Given this, several recent studies have examined whether the rs641738 variant is associated with plasma lipid levels as well as cardiovascular disease. A recent study by Tabassum *et al.* (54) found that the rs8736 SNP near *MBOAT7* not only associates with its predicted metabolic products (38:3 and 38:4 PI) but also associates with the phenotype of venous thromboembolism. This is the only study linking *MBOAT7* to venous thromboembolism to date, but given the significant association (*P* = 1.3 × 10^−3^), this is worth additional investigation. Studies by Brouwers *et al.* (55, 56) have also looked at the association between the rs641738 locus and coronary artery disease across 48 GWAS studies including 60,801 cases and 123,504 controls. Despite a clear link to liver injury, the rs641738 SNP is not associated with coronary artery disease risk regardless of what genetic model is used (55, 56).

As mentioned previously, genetic variants near *MBOAT7* have been linked to liver cancer in subjects with both viral and nonviral backgrounds. However, there is now emerging evidence that MBOAT7 function may also be linked to other malignancies outside the liver. Heinrichs *et al.* (57) performed a genetic fine mapping study in 1,926 gastric cancer patients and 2,012 controls of European descent and found that a novel SNP rs229400 was associated with reduced *MBOAT7* expression and increased risk of gastric cancer. Saliakoura *et al.* (58) found that MBOAT7 substrate and product lipids (LPIs and PIs, respectively) were altered in cell models of non-small cell lung cancer and further showed that *MBOAT7* knockdown suppresses tumorigenesis and improves the survival of mice bearing human cancer xenografts. Neumann *et al.* (59) performed unbiased lipidomics in tumor and nontumor specimens from patients with the common kidney cancer clear cell renal cell carcinoma and likewise found a reciprocal alteration in MBOAT7 substrate and product lipids in the tumor microenvironment. This study also showed that clear cell renal cell carcinoma cell lines that genetically lacked *MBOAT7* decreased their proliferation rate and prevented tumor formation in mouse xenograft studies (59). Although MBOAT7 likely plays unique roles in different cancer types, additional studies are warranted to understand the mechanisms by which MBOAT7 may shape key signaling lipids within the tumor microenvironment.

In addition to the common polymorphism near the *MBOAT7* gene, several homozygous null mutations (Online Mendelian Inheritance in Man: 606048) have been identified in subjects with severe intellectual disability, epilepsy, and autistic phenotypes (60, 61, 62, 63, 64). Johansen *et al.* (60) originally described five distinct variants in coding regions of *MBOAT7*, all of which are predicted to affect protein expression and function, which were associated with intellectual disability in six consanguineous families from Pakistan (Fig. 1B). Several follow-up studies have identified >16 loss-of-function mutations in the human *MBOAT7* gene, most of which are associated with intellectual disability, developmental delay, epilepsy, microencephaly or macroencephaly, and autism-like phenotypes (61, 62, 63, 64). Collectively, these studies suggest that MBOAT7 function is linked to neurodevelopmental issues that likely originate in the central nervous system. Finally, one recent report has also linked MBOAT7 function to coronavirus disease 2019 (COVID-19)-related liver injury (65). Machill *et al.* (65) longitudinally studied a cohort of 44 COVID-19 positive patients and found that the rs641738 T allele was associated with an increased risk of liver injury. Collectively, over the past 6 years, numerous human studies have linked genetic variation in the *MBOAT7* locus to various diseases, prompting several groups to follow up using animal models to establish causal links to liver disease progression and other related phenotypes.

## Loss of *MBOAT7* function in mice promotes liver injury and systemic glucose intolerance

Metabolic phenotyping of the global *Mboat7* knockout mouse is limited by the fact that these mice are viable only for a few weeks following birth. This is due to the critical role that MBOAT7 plays in the brain cortical lamination and neuronal migration (66, 67). To overcome this barrier, we recently generated an in vivo knockdown approach using antisense oligonucleotides (ASOs), which predominately target liver, adipose tissue, and cells within the reticuloendothelial system to selectively knockdown *Mboat7* expression in adult mice (37). This ASO approach allowed us to circumvent the postnatal lethality of global *Mboat7* deletion (66, 67) and permitted the first reported investigation into high-fat diet-induced liver disease progression with near complete loss of function of *Mboat7* in the liver. Helsley *et al.* (37) showed that knockdown of *Mboat7* promotes hepatic steatosis, hepatocyte death, inflammation, and early fibrosis in high-fat diet-fed mice. However, genetic deletion of *Tmc4* did not provoke hepatic steatosis (37), which is an important finding because the human rs641738 SNP lies within exon 1 of the *TMC4* gene (Fig. 1A). This article also demonstrated that hepatic expression of *MBOAT7* is reduced in obese humans and rodents, independent of rs641738 status, and that the expression of *Mboat7* in the liver and adipose tissue of mouse is negatively correlated with obesity and insulin sensitivity across all the strains represented by the Hybrid Mouse Diversity Panel (37). This work further showed that *Mboat7* loss of function promotes striking hyperinsulinemia and hepatic insulin resistance in high-fat diet-fed mice (37). This work also showed that genetic deletion of *MBOAT7* in human hepatoma cell lines can promote triacylglycerol accumulation in a cell autonomous manner by both limited fatty acid oxidation and promoting de novo lipogenesis (37). ASO-mediated *Mboat7* knockdown also promoted liver-specific accumulation of Mboat7 substrate lipids (saturated and monounsaturated LPIs) and showed that direct administration of LPI lipids can rapidly induce hepatic inflammatory and fibrotic gene expression programs in an *Mboat7*-dependent manner in mice. Within a year of this initial report in October 2019 (37), four independent articles using different approaches to limit Mboat7 activity in the liver were published, further bolstering the concept that *Mboat7* loss of function contributes to liver injury.

In early 2020, Meroni *et al.* published work using a similar in vivo *Mboat7* knockdown approach and found strikingly similar findings to those reported by Helsley *et al.* (38). Using morpholino oligonucleotides (MPOs) to knockdown the expression of *Mboat7*, Meroni *et al.* (38) showed that *Mboat7* loss of function promoted steatosis in mice. This work also showed that hepatic mRNA and proteins levels of *Mboat7* are suppressed in mouse models of NAFLD/NASH including the methionine choline-deficient diet as well as in leptin-deficient (*ob/ob*) mice. Furthermore, Meroni *et al.* (38) showed that acute treatment with the key anabolic hormone insulin can suppress both the mRNA and protein expression of *MBOAT7* suggesting a potential role for *MBOAT7* in insulin action. Another interesting finding in this work was that in hepatoma cells lacking *MBOAT7*, there was an apparent overexpression of key lipid transporter called fatty acid transport protein 1 (FATP1) (38). The group went on to show that heterozygous genetic deletion of *FATP1* was able to rescue the increased level of lipogenic gene expression seen in *MBOAT7*-deficient cells (38). Collectively, the work by Meroni *et al.* (38) bolsters the idea that *MBOAT7* expression is downregulated in mouse models of obesity (*ob/ob*) or NAFLD/NASH progression (methionine choline-deficient diet) and confirms that *Mboat7* knockdown in mice promotes hepatic steatosis.

Following up on these ASO and MPO knockdown studies (37, 38) in 2021, three independent laboratories generated hepatocyte-specific *Mboat7* knockout mouse lines to better understand the cell autonomous role for MBOAT7 in liver disease progression. First, Tanaka *et al.* (39) demonstrated that hepatocyte-specific genetic deletion of *Mboat7* (*Mboat7*^HKO^) was sufficient to promote hepatic steatosis, and when challenged with a high-fat diet, *Mboat7*^HKO^ mice developed early fibrosis. This work also showed that deletion of *MBOAT7* in a human hepatic spheroid culture promoted triacylglycerol accumulation and collagen deposition (39). An extremely important finding from the work of Tanaka *et al.* was that the hepatic steatosis seen with *MBOAT7* deficiency was due in part to enhanced triglyceride synthesis through a noncanonical pathway. Using radiolabeled glycerol tracers, it was shown that glycerol incorporation into PI was selectively increased in *MBOAT7*-deficient cells, whereas other major phospholipid classes such as phosphatidylcholine (PC) and phosphatidylethanolamine (PE) were unaffected (39). To follow up, this group found that the expression and activity of an enzyme called CDP-diacylglycerol synthase 2 (CDS2), which converts phosphatidic acid (PA) to CDP-diacylglycerol (CDP-DAG), was increased in *MBOAT7*-deficient cells (39). Therefore, the authors suggested that the overall reduction in acyl chain remodeling seen in MBOAT7-deficient cells promoted an abnormally high PI turnover via phospholipase C activity and at the same time have overactive CDS2 activity to promote overproduction of CDP-DAG, which is a critical precursor for triacylglycerol biosynthesis.

Like the work of Tanaka *et al.*, Thangapandi *et al.* (40) generated an independent line of *Mboat7*^HKO^ mice, but instead of challenging them with a high-fat diet (which only promotes early fibrosis), they chose a profibrotic diet known as the high fat, methionine low, choline-deficient diet. When challenged with this fibrosis-inducing diet, *Mboat7*^HKO^ mice displayed increased levels of profibrotic gene expression, increased hydroxyproline levels, and in some cases, bona fide bridging fibrosis that was not found in control mice (40). Like other mouse *Mboat7* loss-of-function studies (37, 38, 39), this study also found reductions in the MBOAT7 product (38:4 PI) and striking increases in its substrate LPIs (16:0 LPI and 18:0 LPI) in the liver (40). This article also confirmed that the rs641738 T allele is associated with lower 38:4 in liver biopsies from humans (40). Finally, a third *Mboat7*^HKO^ mouse model was generated by Xia *et al.* (41) and studied under chow-fed condition. Much like the other *Mboat7* loss-of-function studies, this work also saw striking hepatic steatosis in *Mboat7*^HKO^ mice. However, the work was the first to collect liver tissue under well-defined fasting and refeeding conditions to examine alterations in refeeding-induced alterations in hepatic metabolism. Following 3 h of refeeding, *Mboat7*^HKO^ mice showed a marked increase in de novo lipogenesis driven by activation of the master transcriptional regulator SREBP-1c. In a simple yet elegant follow-up study, Xia *et al.* (41) then crossed *Mboat7*^HKO^ mice to mice lacking SREBP cleavage-activating protein (*Scap*) and found that the hepatic steatosis observed in *Mboat7*^HKO^ mice is not evident in *Mboat7*^HKO^ mice that fail to activate the SREBP transcription factors (i.e., *Mboat7*^HKO^ mice *+ Scap*^−/−^). This work shows that SREBP-1c-driven de novo lipogenesis programs are in part necessary for the hepatic steatosis seen with *MBOAT7* deficiency. Yet, it still remains unclear how MBOAT7-driven PI remodeling can impact SREBP processing and transcriptional activation. Collectively, all mouse studies to date agree that *Mboat7* loss of function in hepatocytes is sufficient to promote lipid accumulation in the liver (37, 38, 39, 40, 41). Although some proposed mechanisms behind this have been put forth, we still do not completely understand how defective MBOAT7-driven PI remodeling can promote the progression from simple steatosis to NASH, fibrosis, cirrhosis, or even HCC. As the search for mechanistic links continues, there are clear lessons that can be learned from the unique biochemical role that MBOAT7 plays in the remodeling of PI lipids.

## Lessons learned from MBOAT7 biochemistry: diverse roles of MBOAT7 substrate and product lipids in signal transduction and metabolic homeostasis

**Fig. 2:**
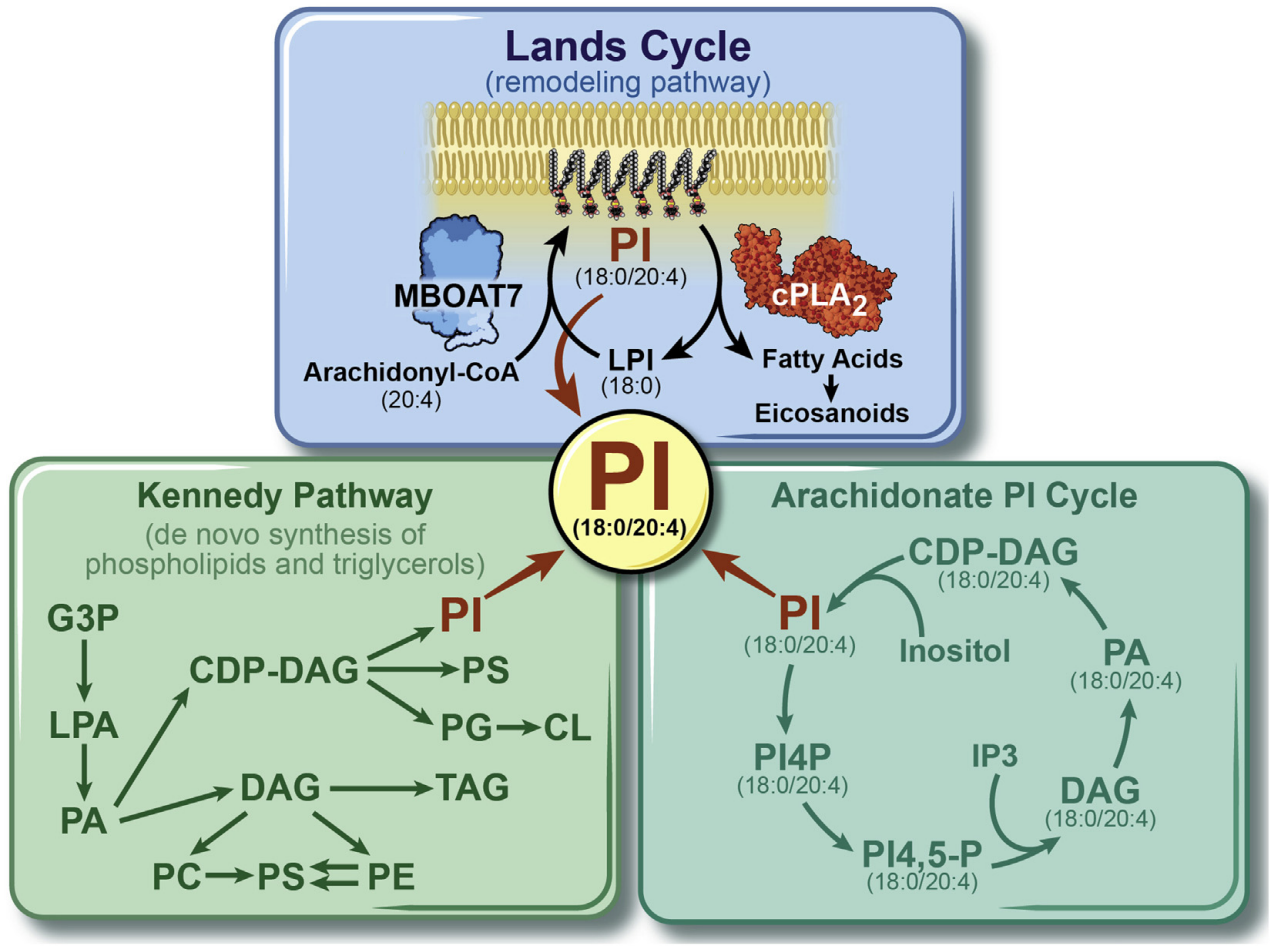
MBOAT7 plays a unique role in PI homeostasis by esterifying LPI to several PUFA acyl-CoAs including arachidonyl-CoA (20:4) to generate the most abundant PI species (PI 38:4; where the *sn*-1 position harbors a stearate 18:0 and the *sn*-2 position harbors arachidonic acid 20:4). Within the Land’s cycle, MBOAT7-dependent esterification opposes the actions of cytosolic PLA_2_, which instead cleaves PUFAs such as AA from 38:4 PI to generate downstream AA-derived lipid mediators including leukotrienes, prostanoids, thromboxanes, lipoxins, and epoxyeicosatrienoic acids. In parallel to MBOAT7-driven synthesis of 38:4 PI, the de novo “Kennedy” pathway can also generate large amounts of PI 38:4 using CDP-DAG as a substrate. Although MBOAT7 specifically regulates reacylation in the Land’s cycle, it could indirectly impact the substrate availability of 38:4 PI to the arachidonate PI cycle, which is initiated by a parallel pathway where inositol is added to CDP-DAG (18:0/20:4) by phosphatidylinositol synthase (PIS) to also form 38:4 PI. Current evidence suggests that both PIS-generated as well as MBOAT7-generated 38:4 can serve as substrate for phosphatidylinositol kinases to form the key second messengers known as PIPs (including PI [18:0/20:4]-4P, PI [18:0/20:4]-4,5P_2_) and lipid mediators downstream of phospholipase C in the arachidonate PI cycle (IP3, DAG [18:0/20:4], PA [18:0/20:4], and CDP-DAG [18:0/20:4]). CL, cardiolipin; G3P, glycerol-3-phosphate; IP3, inositol trisphosphate; LPA, lysophosphatidic acid, PG, phosphatidylglycerol; PIS, phosphatidylinositol synthase; PI4P, phosphatidylinositol-4-phosphate; PI4,5-P, phosphatidylinositol 4,5-bisphosphate; PS, phosphatidylserine.

Although there are only a handful of articles examining the biochemistry of MBOAT7, these studies provide important clues into its broader role in physiology and disease. MBOAT7 has been biochemically annotated as a lysophospholipid acyltransferase enzyme (LPI acyltransferase 1) that preferentially esterifies LPI lipids to arachidonyl-CoA to form the major PI species (38:4) in the inner leaflet of cell membranes (68, 69, 70, 71) (Fig. 2). MBOAT7 is a unique contributor to the Lands’ cycle, which is a series of deacylation reactions catalyzed by phospholipase and reacylation reactions driven by lysophospholipid acyltransferases that synergize to alter phospholipid fatty composition, creating membrane diversity and asymmetry (72, 73). In a series of landmark articles in the 1950s and beyond William E.M. Lands discovered that fatty acid composition of phospholipids at the *sn*-1 and *sn*-2 positions are dynamically shaped by the classic Kennedy “de novo” pathway (74) as well as the pathway holding his namesake (Fig. 2). The Land’s cycle is initiated when phospholipases, most prominently phospholipase A_2_ (PLA_2_), cleave fatty acids from the *sn*-2 position, and then lysophospholipid acyltransferases such as MBOAT7 can selectively re-esterify new PUFAs in that position to complete the remodeling cycle (72, 73) (Fig. 2). It is important to note that, unlike other lysophospholipid acyltransferases, MBOAT7 only diversifies the fatty acid composition of membrane PI species and not phospholipids with other head groups (PA, PC, phosphatidylserine, PE, phosphatidylglycerol, etc) (72, 73). This substrate specificity has been seen in cell-based biochemical studies (68, 69, 70, 71) and also confirmed in *Mboat7* loss of function in mice (37, 38, 39, 40, 41). Given the highly selective role of MBOAT7 in acylating LPI lipid substrates to form product PI, our discussion here focuses on how these inositol-containing lipids play extremely important roles in different aspects of signal transduction, membrane shape and fusion events, and other cellular functions.

*MBOAT7* is the mammalian ortholog of *mboa-7*, which was originally identified in a genetic screen in *Caenorhabditis elegans* (66). Genetic deletion of *mboa-7* in *C. elegans* resulted in highly specific depletion of arachidonic acid (AA)- and EPA-containing PI but not PC or PE (66). In a series of elegant studies, Gijón *et al.* (68, 69) showed that human MBOAT7 has a similar substrate preference. First, they expressed human *MBOAT7* in yeast lacking the major yeast lysophospholipid acyltransferase Ale1p and found that human MBOAT7 was highly selective for arachidonyl-CoA (68). However, long-chain PUFAs were not assayed in this study. Gijón *et al.* went on to show that the drug thimerosal could effectively block the acyltransferase activity of MBOAT7 and that MBOAT7 is the major contributor to Land’s cycle remodeling of PI lipids in human neutrophils (68, 69). More recent work by Caddeo *et al.* has shown that human MBOAT7 can use both arachidonyl-CoA as well as eicosapentaenoyl-CoA as substrates and described an active-site dyad composed of asparagine (Asn-321) and histidine (His-356) facilitating catalysis (70). This same group also used computational and experimental approaches to predict that MBOAT7 likely has six membrane-spanning domains, and that active site histidine (His-356) has a lumenal facing orientation (71). It is important to note that although the current biochemistry studies support a role for MBOAT7 in preferentially acylating 18:0 LPI with either arachidonyl-CoA or eicosapentaenoyl-CoA as substrates, which would generate the 38:4 and 38:5 PI species, respectively, we and others have also seen that *Mboat7* loss of function in mice also results in a significant reduction in the 38:3 PI species as well (37, 41). This 38:3 PI species is predicted to have stearic acid (18:0; *sn-1*) and di-homo-γ-linolenic acid (DGLA; 18:3, *sn*-2), so it remains possible that DGLA can also serve as a metabolic substrate; although this has never been formally tested. Given that MBOAT7 can utilize AA, EPA, and DGLA CoAs as potential substrates, it will be important to consider substrate availability in future studies because no studies to date have provided exogenous dietary sources of EPA or DGLA under conditions of MBOAT7 deficiency. It is interesting to note that a recent large population study by Mann *et al.* (75) showed that the rs641738 SNP significantly correlates with plasma levels of free DGLA.

### So the key question still remains

How does MBOAT7 loss of function result in liver injury or other human diseases? The most straightforward explanation is that either the substrates (LPIs or fatty acyl-CoAs) or products (38:3, 38:4, and 38:5 PI) of the MBOAT7 reaction initiate some signal in the liver to promote hepatic steatosis, inflammation, and fibrosis. One obvious potential mechanism by which MBOAT7 could influence hepatic inflammation is due to its central role in AA incorporations into phospholipids. As mentioned previously, within the Land’s cycle, MBOAT7 opposes the actions of PLA_2_ enzymes (Fig. 2), which work to liberate free AA from PI and other phospholipids to provide substrates for downstream cytochrome P450-, cyclooxygenase-, lipoxygenase-driven production of AA-derived lipid mediators, including leukotrienes, prostanoids, thromboxanes, lipoxins, and epoxyeicosatrienoic acids (72, 73, 74, 75, 76). However, when greater than 20 molecular species of AA-derived lipid mediators have been measured in the liver of *Mboat7* loss-of-function mice, three independent studies consistently showed no alterations compared with control mice (37, 39, 40). This strongly suggests that *Mboat7* deficiency does not quantitatively contribute to the hepatic levels of AA-derived lipid mediators under diverse dietary conditions, making it highly unlikely that this is the reason MBOAT7 loss of function promotes liver injury.

Another plausible explanation could be that reductions in MBOAT7 products (38:3 PI, 38:4 PI, or 38:5 PI) may elicit key cellular changes that promote liver injury. In all tissues, the most abundant PI species is detected at *m/z* 885.5494, where the negative ion corresponds to 38:4 PI. Given its relative abundance in cells, compared with other minor PI species, it is reasonable to assume that the ∼30% reduction in hepatic 38:4 PI (37, 38, 39, 40, 41) may in part play a role in liver injury. Although MBOAT7 specifically regulates reacylation in the Land’s cycle, it could indirectly impact the substrate availability of 38:4 PI to the arachidonate PI cycle (Fig. 2). The arachidonate PI cycle is thought to be initiated by a parallel pathway in the endoplasmic reticulum where inositol is added to CDP-diacylglycerol (18:0/20:4) by phosphatidylinositol synthase to also form 38:4 PI. It remains possible that both phosphatidylinositol synthase-generated as well as MBOAT7-generated 38:4 can serve as a substrate for PI kinases to form the key second messengers known as phosphatidylinositol phosphates (PIPs including PI [18:0/20:4]-4P, PI [18:0/20:4)-4,5P_2_], and lipid mediators downstream of phospholipase C in the arachidonate PI cycle (IP3, DAG [18:0/20:4], PA [18:0/20:4], and CDP-DAG [18:0/20:4]) (Fig. 2). An important study by Anderson *et al.* (67) quantified PIP species in the liver of global *Mboat7*^−/−^ mice and found that total PIPs, PI (18:0/20:4)-4P, and PI (18:0/20:4)-4,5P_2_ were significantly reduced in *Mboat7*^−/−^ mice. It is important to note that >85% of PIP species in cultured cells have an *sn*-1 18:0 and *sn*-2 20:4 acyl chain composition (i.e., in part originate from the MBOAT7 product 38:4 PI) (77, 78). These alterations in PIP signaling lipids seen with *Mboat7* deficiency could have important consequences in cellular signal transduction, given that PIPs are common second messengers generated downstream of ligand activation of numerous receptor systems, including hormone, growth factor, cytokine, and chemokine receptors (79, 80, 81, 82). PIPs also play diverse roles in shaping protein-lipid interactions, membrane fusion events, vesicular transport, solute channel function, and cytoskeletal arrangement (79, 80, 81, 82). Also, given the role of anionic lipids in controlling membrane dynamics, PIP lipids are involved in nearly all steps of autophagy including initiation of autophagosome biogenesis and autophagosome-lysosome fusion (83, 84). In addition to alterations in AA-containing PIPs, alterations in 38:4 PI itself could potentially influence cellular function given the fact that anionic phospholipids like 38:4 PI can influence biophysical properties of cell membranes and membrane curvature in part by altering the membrane electrostatic fields (85, 86). Collectively, given the fact that MBOAT7 generates the most abundant species of PI (38:4), and key cellular PIPs (PI [18:0/20:4]-4P and PI [18:0/20:4]-4,5P_2_), there is a strong potential that this could alter cellular signal transduction, protein-lipid interactions, membrane fusion events, vesicular transport, solute channel function, cytoskeletal arrangement, and autophagic flux. However, each of these hypotheses will need to be tested with biochemical and cell biology tools appropriate for each possibility.

Another potential way that MBOAT7 loss of function could promote liver injury is by the abnormal accumulation of substrate LPI lipids in the liver, which has been seen in several mouse studies (37, 38, 40). Several lysophospholipids such as lysophosphatidic acid and sphingosine-1-phosphate have been repeatedly shown to be potent regulators of inflammatory diseases (80, 87). These bioactive lysophospholipids act primarily by binding to dedicated cell surface receptors in the G protein-coupled receptor family to elicit cellular signaling responses (80, 87, 88). Although a plethora of research has been conducted with lysophosphatidic acid and sphingosine-1-phosphate, there is emerging evidence that LPI lipids can also serve as relevant lipid signals promoting proinflammatory, profibrotic, and endothelial-activating effects (37, 89, 90, 91, 92, 93, 94, 95, 96, 97, 98, 99, 100, 101, 102). Several recent articles have shown that the MBOAT7 substrate LPIs (16:0, 18:0 LPI, and 18:1 LPI) can initiate rapid signaling processes in macrophages and endothelial cells to promote inflammatory cytokine production as well as endothelial cell activation (89, 90, 91, 92, 93, 94, 95, 96, 97, 98, 99, 100, 101, 102). Given that ASO-mediated knockdown of *Mboat7* resulted in the accumulation of LPI lipids in the liver (37, 38, 40), Helsley *et al.* followed up on this observation to demonstrate that only two intraperitoneal doses of exogenous 18:0 LPI or 18:1 LPI were able to stimulate proinflammatory and profibrotic gene expression in the liver in *Mboat7* knockdown mice but not in control mice where MBOAT7 activity is sufficient to esterify the exogenous LPI (37). Collectively, these results show that LPI lipids can promote inflammatory pathways in macrophages and endothelial cells, and the ability of LPI lipids to signal may be shaped by MBOAT7 function (i.e., MBOAT7-driven esterification of LPIs renders them unable to signal normally). LPI lipids are ligands for G protein-coupled receptor 55 (GPR55), and there have been consistent findings showing that p38 mitogen-activated protein kinase is a critical downstream effector of LPI-GPR55 signaling (88, 91, 97, 99). It is interesting to note that the LPI receptor, GPR55, has already been pursued as a potential drug target in obesity and insulin resistance, and selective agonists and antagonists have been synthesized with mixed results (95, 96). In addition, it is important to note that a recent report by Fondevila *et al.* (102) showed that the expression of the LPI receptor GPR55 is increased in human and mouse models of NAFLD/NASH, and that LPI lipids can promote hepatic steatosis in a GPR55-dependent manner. This work provides provocative new evidence that activation of the LPI-GPR55 system is associated with human NAFLD progression in a strikingly similar manner to that seen with *Mboat7* deficiency (i.e., a condition where LPI lipids accumulate) (37, 38, 39, 40, 41). Although there is a growing body of evidence that LPIs signal through the GPR55 receptor, there is also evidence of non-GPR55-dependent signaling so the search for additional LPI receptors is warranted. If the LPI-GPR55 system can be consistently linked to liver injury, it will be exciting to test whether GPR55 antagonists can provide therapeutic benefit in ALD, NAFLD, or viral hepatitis. At this point, our understanding of LPI signaling is still immature, and additional work is needed to fully realize areas of potential therapeutic intervention.

## Concluding remarks

**Fig. 3:**
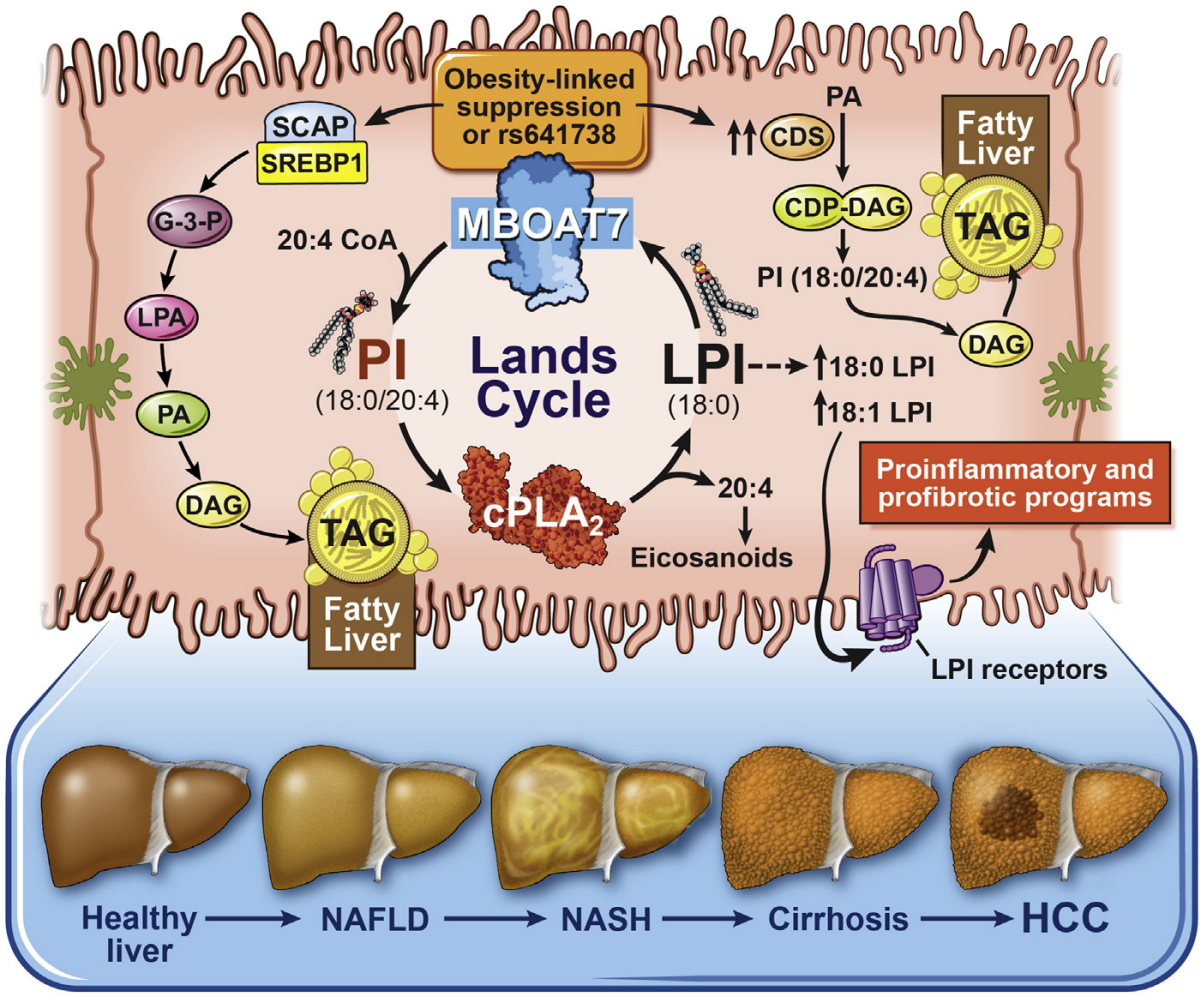
Working model of our current understanding of the mechanisms by which MBOAT7 loss of function promotes liver disease progression. Either the common rs641738 (C>T) SNP or obesity/hyperinsulinemia can reduce the levels of MBOAT7 expression and enzymatic activity. When MBOAT7 function is suppressed, there is abnormal storage of triacylglycerol (TAG) in the liver (i.e., hepatic steatosis) via *1*) activation of the SCAP-SREBP-1c pathway to promote canonical de novo lipogenesis; *2*) (41) activation of noncanonical pathway of lipid synthesis via upregulation of CDS2 (39), or *3*) the fatty acid transporter FATP1 is overexpressed facilitating lipid deposition (38). In addition, the accumulation of MBOAT7 substrate LPIs can promote steatosis (100, 102) and proinflammatory and profibrotic signaling under conditions where MBOAT7 function is diminished (37). Collectively, when MBOAT7 function is diminished, there is an imbalance in both the substrates (LPIs, fatty acyl-CoAs, or free fatty acids) and products (38:3, 38:4, and 38:5 PI and phosphorylated versions of these PI lipids) of the MBOAT7 reaction, which likely work in concert in the liver to promote the progression of liver disease from hepatic steatosis toward inflammation and fibrosis. cPLA_2_, cytosolic PLA_2_; G3P, glycerol-3-phosphate; LPA, lysophosphatidic acid.

Since the original GWAS study by Buch *et al.* in December 2015 linking the rs641738 SNP near *MBOAT7* to liver disease, there has been tremendous progress in our understanding of how *MBOAT7* is mechanistically linked to the progression of ALD-, NAFLD-, and viral-driven cirrhosis and HCC. Although not all human studies agree there is a uniform association between the rs641738 SNP with liver injury across all etiologies, all studies in mice confirm that *Mboat7* loss of function in mice can promote hepatic steatosis and fibrosis (37, 38, 39, 40, 41). Based on our current understanding, the working model is that *MBOAT7* deficiency (either via rs641738-driven or obesity-related suppression) can promote steatosis via *1*) activation of the SCAP-SREBP-1c pathway to promote canonical de novo lipogenesis, *2*) activation of a noncanonical pathway of lipid synthesis via upregulation of CDS2, or *3*) the fatty acid transporter FATP1 is overexpressed facilitating lipid deposition (Fig. 3). In addition, the accumulation of MBOAT7 substrate LPIs can promote steatosis (100, 102) and proinflammatory and profibrotic signaling under conditions where *MBOAT7* function is diminished (37) (Fig. 3). Although we have gained some mechanistic understanding of how *Mboat7* loss of function can promote steatosis in mouse models (Fig. 3), there are still many unanswered questions that remain. For instance, given the mixed results in human genetic association studies (some showing a significant association between rs641738 and liver injury and some studies showing no association), it is unclear why the findings are study dependent. The inconsistencies in the human association studies may be explained in part by the ability of obesity and/or hyperinsulinemia to suppress *MBOAT7* mRNA and protein levels (37, 38), which appears to be independent of and in addition to the rs641738-mediated suppression of *MBOAT7* expression (37). Not all studies to date have looked at confounding factors such as obesity or circulating insulin levels.

As additional human studies are performed, it will also be critical to ensure they are properly powered to detect predicted associations but maybe even more importantly, it may be critical to consider dietary patterns. The key question here is whether the MBOAT7 substrate fatty acids such as AA, EPA, and DGLA being consumed, and whether liver disease phenotypes can be shaped by substrate availability. Emerging evidence suggests that MBOAT7 can utilize acyl-CoA substrates of AA (20:4, n-6), EPA (20:5, n-3), and DGLA (20:3, n-6) (37, 38, 39, 40, 41, 66, 68, 69, 70, 71), yet most rodent diets have extremely low levels of EPA and DGLA. Future animal studies should examine the ability of *Mboat7* depletion to drive liver injury under controlled dietary settings with sufficient levels of AA, EPA, or DLGA. Likely, PLA_2_-driven products originating from the Land’s cycle (where MBOAT7 will oppose and re-esterify) will be different if the major PI *sn*-2 species AA is replaced by EPA or DGLA. It is extremely important to remember when one is studying a PUFA-metabolizing enzyme, that substrate availability is key to shaping the downstream metabolic products that can either be largely proinflammatory (as is the case for AA) or anti-inflammatory (as is the case for EPA).

Another extremely important unanswered question is: how does MBOAT7 activity alter the proteolytic processing of the master transcriptional regulation of de novo lipogenesis SREBP-1c? Could MBOAT7 substrate or product lipids impact SREBP-1c processing? Alternatively, given that both MBOAT7 and SREBP-1c can reside in the endoplasmic reticulum, could there be a noncatalytic role that MBOAT7 plays in the SCAP-INSIG-SREBP-1c axis? Aligned with this, how does MBOAT7 loss of function promote CDS2-dependent generation of CDP-DAG to promote fatty liver? Another key question that has come from mouse studies is how to reconcile the fact that either ASO- or MPO-mediated silencing of *Mboat7* (which can target multiple cell types) promotes profound hyperinsulinemia and systemic insulin resistance (37, 38). However, hepatocyte-specific deletion of *Mboat7* does not alter glucose or insulin homeostasis in multiple studies (39, 40, 41). It is reasonable to assume that the abundant expression of Mboat7 in adipose tissue (37) or myeloid cells (68, 69) may play a role in systemic insulin resistance, but additional studies are required to formally test this hypothesis. Given that floxed mice are available, it will also be interesting to test the cell autonomous roles of *Mboat7* in nonparenchymal cells in the liver and also in the central nervous system as it relates to cortical lamination. As mechanism of action studies continue, it will be important to consider where MBOAT7 substrates (LPIs or fatty acyl-CoAs) or products (38:3, 38:4, and 38:5 PIs or PIPs) are localized within cells, as it is well known that each of these diverse lipids have distinct subcellular localization, which determines downstream structural or signaling functions. As research advances in this area, it is exciting to envision translational potential in NAFLD/NASH, viral hepatitis, and potentially even COVID-19-related liver disease. Given the clear human genetic association, and a causal link to liver disease progression in animals, there is tremendous untapped therapeutic potential within the LPI-MBOAT7-PI axis. This is yet another example of how human genetics can powerfully identify new pathways relevant to human disease and further supports the long-standing notion that abnormal lipid metabolism drives liver injury. Moving forward, we simply need a village of creative lipid scientists to identify the mechanism(s) by which MBOAT7 loss of function promotes human disease, with the long-term goal of developing new treatments for diverse liver diseases.

## Data availability

There are no data in this review article.

## Conflict of interest

The authors declare that they have no conflicts of interest with the contents of this article.
